# Pre-pregnancy body mass index, weight gain and energy intake in pregnant women with gestational diabetes mellitus

**DOI:** 10.1186/1758-5996-7-S1-A79

**Published:** 2015-11-11

**Authors:** Renata Selbach Pons, Fernanda Camboim Rockett, Bibiana de Almeida Rubin, Maria Lúcia Rocha Oppermann, Vera Lúcia Bosa

**Affiliations:** 1 Universidade Federal do Rio Grande do Sul, Porto Alegre, Brazil

## Background

Studies have shown that maternal obesity is associated with increased risk of Gestational Diabetes Mellitus (GDM). When compared to normal weight women, the chance of developing the disease steadily increases in those who are overweight, obese and severely obese. Excessive gestational weight gain is also associated with risk of GDM. Moreover, dietary factors, before and during pregnancy, have been linked to increased risk for GDM. Nutritional intervention is important for the control of GDM, as it provides benefits to mother and fetal health. However, the literature remains inconclusive on the role of dietary factors in the development of this disease.

## Objective

To evaluate and relate pre-pregnancy body mass index (PPBMI), weight gain and energy intake at diagnosis of GDM.

## Materials and methods

Cross-sectional study of 76 pregnant women with GDM referred to a multidisciplinary clinic for high risk pregnancies in a tertiary hospital in southern Brazil. A questionnaire gathered sociodemographic, clinical, anthropometric and lifestyle data, and also a Food Frequency Questionnaire was calculated in order to measure food and energy intake. Pre-pregnancy nutritional status and weight gain were classified according to guidelines by the Institute of Medicine. Energy recommendations were calculated according to the Dietary Reference Intakes. Pearson Chi-square test, analysis of variance, Kruskal-Wallis and Bland-Altman tests were employed.

## Results

**Figure 1:**
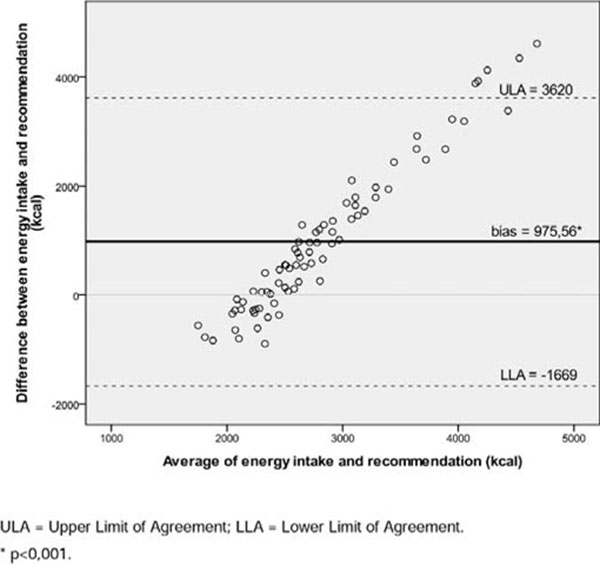
Graphical analysis on Bland-Altman showing agreement between the average of energy intake and recommendation (kcal) in a sample of pregnant women at diagnosis of gestational diabetes mellitus. Rio Grande do Sul/Brazil, 2014.

**Figure 2:**
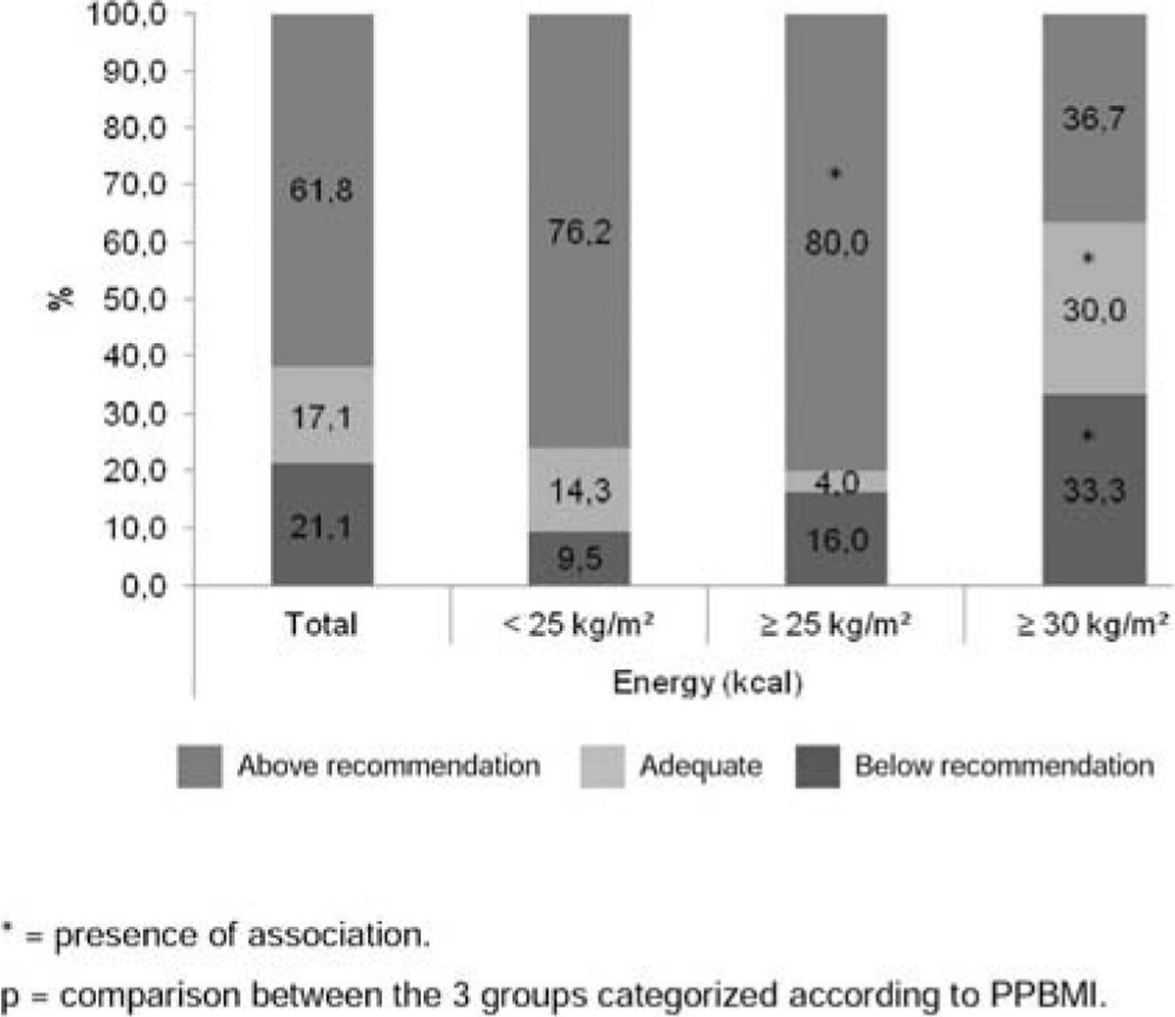
Adequacy of energy intake in total sample and stratified by the pre-pregnancy body mass index (PPBMI) classification in a sample of pregnant women at diagnosis of gestational diabetes mellitus. Rio Grande do Sul/Brazil, 2014.

We evaluated pregnant women aged 20 to 46 yrs. old. Thirty-eight percent were over 35 yrs. old. We found a high prevalence of pre-pregnancy overweight/obesity (n=55; 72.3%) and excessive gestational weight gain (Table 1). Average energy intake was above recommendations (p<0.001) (Figure 1). Women with PPBMI <25 kg/m^2^ or ≥25 kg/m^2^ experienced greater weight gain than the obese group (p=0.002) (Table 1). Women with PPBMI 25.0-29.9 kg/m^2^ had higher energy intakes than is recommended (p=0.006) (Figure 2).

**Table 1:** Anthropometric data of the total sample and stratified by the classification of pre-pregnancy Body Mass Index (PPBMI) in a sample of pregnant women at diagnosis of Gestational Diabetes Mellitus, Rio Grande do Sul/Brazil, 2014. [Mean±SD or Median (interquartile range) or n (%)]

			PPBMI (kg/m^2^)		p**
	**Total** (n=76)	**< 25** (n=21)	**≥ 25** (n=25)	**≥ 30** (n=30)	
		
Feature					
**Weight gain before diagnosis (kg)**	8,1 (2,2 – 11,1)	9.0 (7,6-11,1)^a^	9,2 (2,5-13,9)^a^	3,4 (0,1-9.0)^b^	0.002
*Below the recommended*	26 (34,2)	4 (19.0)	7 (28.0)	15 (50.0)	0,166
*Adequate*	4 (5,3)	2 (9,5)	1 (4.0)	1 (3,3)	
*Above the recommended*	46 (60,5)	15 (71,4)	17 (68.0)	14 (46,7)	
**BMI at diagnosis (kg/m^2^)**	32,2±5,8	26,1±2.0^a^	30,9±3,1^b^	37,5±4,5^c^	< 0.001
*Low weight*	1 (1,3)	1 (4,8)	0 (0)	0 (0)	< 0.001
*Normal weight*	16 (21,1)	14 (66,7)*	2 (8.0)	0 (0)	
*Overweight*	22 (28,9)	6 (28,6)	14 (56.0)*	2 (6,7)	
*Obesity*	37 (48,7)	0 (0)	9 (36.0)	28 (93,3)*	

a, b, c=mean/median significantly different between groups

*=association found

p**=comparison between groups

BMI=Body Mass Index

## Conclusion

The importance of nutritional counseling before conception and during pregnancy should be emphasized in order to encourage proper PPBMI and avoidance of excessive gestational weight gain, since both are related to risk for GDM. Further studies are needed to better elucidate the role of dietary factors and specific nutrients in the risk for this disease.

